# The Next Frontier in Health Disparities—A Closer Look at Exploring Sex Differences in Glioma Data and Omics Analysis, from Bench to Bedside and Back

**DOI:** 10.3390/biom12091203

**Published:** 2022-08-30

**Authors:** Maria Diaz Rosario, Harpreet Kaur, Erdal Tasci, Uma Shankavaram, Mary Sproull, Ying Zhuge, Kevin Camphausen, Andra Krauze

**Affiliations:** 1 Center for Cancer Research, National Cancer Institute, NIH, Building 10, Bethesda, MD 20892, USA; 2 School of Medicine, Universidad Central del Caribe, Bayamon, PR 00960, USA

**Keywords:** health disparities, sex differences, glioma, genomics, proteomics, large-scale data

## Abstract

Sex differences are increasingly being explored and reported in oncology, and glioma is no exception. As potentially meaningful sex differences are uncovered, existing gender-derived disparities mirror data generated in retrospective and prospective trials, real-world large-scale data sets, and bench work involving animals and cell lines. The resulting disparities at the data level are wide-ranging, potentially resulting in both adverse outcomes and failure to identify and exploit therapeutic benefits. We set out to analyze the literature on women’s data disparities in glioma by exploring the origins of data in this area to understand the representation of women in study samples and omics analyses. Given the current emphasis on inclusive study design and research, we wanted to explore if sex bias continues to exist in present-day data sets and how sex differences in data may impact conclusions derived from large-scale data sets, omics, biospecimen analysis, novel interventions, and standard of care management.

## 1. Introduction

Sex differences are increasingly being explored and reported in oncology and glioma. As potential therapy-, outcome-, and practice-altering sex differences are uncovered, existing gender and gender intersectional-derived disparities mirror the data generated in retrospective and prospective trials, real-world large-scale data sets, and bench work involving animals and cell lines. Women—especially those of child-bearing years—have been excluded from clinical trials to protect them and their fetuses from potential adverse effects and, at times, due to concerns about the inability to control for women’s variable hormonal status [1]. The impact of disparities at the data level is wide-ranging, including female patients receiving treatment based on results of studies generated by a more significant proportion of male participants. Lack of analysis aimed at sex-specific biologic differences can result in potentially unanticipated adverse events secondary to sex-specific differences in disease patterns, metabolism, and drug pharmacokinetics and clearance [2], in addition to clinical differences such as performance status and comorbidities [3], with a secondary inability to potentially capture potential benefits of interventions whose success may hinge on leveraging sex differences.

Given that male vs. female tumor incidence in glioma varies by tumor subtype, region, and age, with males exhibiting a 20–40% higher incidence of CNS (Central Nervous System) tumors in young adults [4], and with treatment received intersecting with risk factors for death, gender [5], and age [6,7], significant intersectionality is expected when analyzing disparities and unbalanced data sets. In the context of glioma, several papers have explored biological factors and sex-dependent differences between men and women and implications related to histology, sex hormones [8,9], pregnancy, menstruation, menopause, and oral contraceptives. There is, however, an ongoing lack of in-depth understanding of the physiology and metabolism that underpins sex differences in glioma, with data just emerging [3,8,10,11,12,13,14,15,16,17]. These biological differences can result in differences in the clinical outcomes of novel interventions. Currently, external validity is evolving and is often lacking for preclinical [18] and clinical data, with data sets and biospecimen repositories yet to be developed. Given this information, a complete understanding of relevant, potentially therapeutically engaging sex differences is lacking. It is therefore essential to understand relevant differences to appropriately conduct a risk assessment and design safe and effective treatments.

We analyzed the literature on women’s data disparities in glioma. To achieve this goal, we aimed to (1) explore the origins of data in this area to understand the representation of women in study samples; (2) identify if sex bias continues to exist in present-day glioblastoma clinical trials by examining how studies are representing the population that may be impacted by novel interventions and standard of care management given current guidelines for study design and analysis aiming for diversity and inclusion; and (3) determine if existing data sets allow for meaningful biospecimen and omics analyses aimed at identifying sex differences. Our hypothesis for this review was that clinical trials generating prospective data and existing retrospective data repositories mirror data in omic and biospecimen-based analyses. As a result, we wanted to determine whether gender annotation and gender-based analysis are reflected in data captured and analyzed, and how this may allow for meaningful future conclusions.

## 2. Examining Prospective and Retrospective Literature Regarding Male and Female Representation

**Figure 1:**
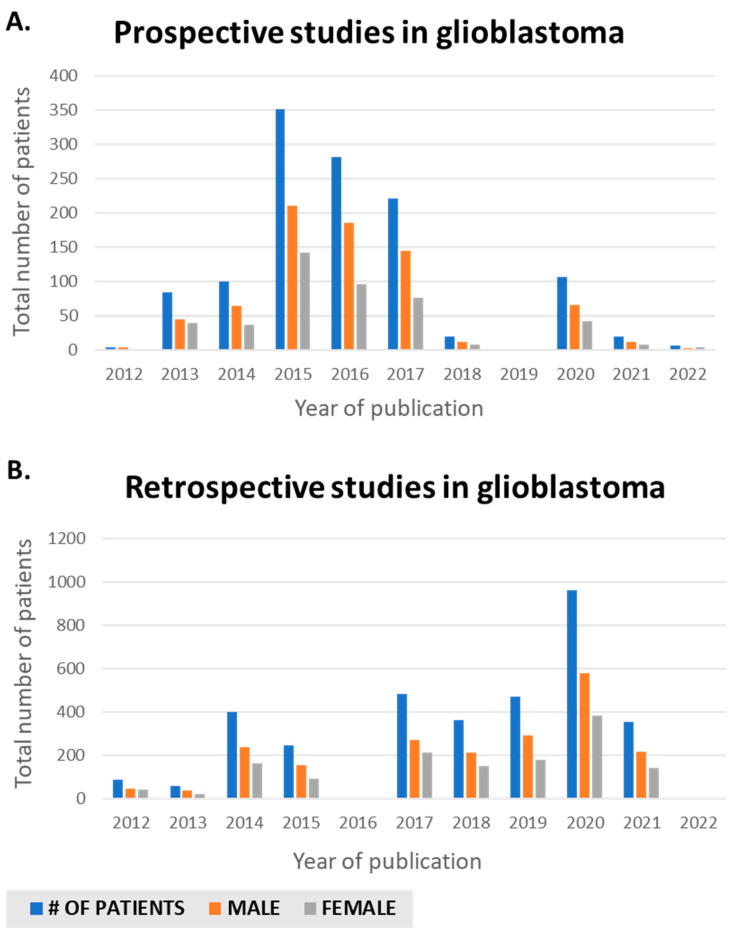
Sex differences publications in glioblastoma in PubMed from 2012 to present. (**A**) Retrospective. (**B**) Prospective.

A literature search was conducted on PubMed using relevant MeSH terms: glioblastomas, gliomas, genomics, and clinical trials. Papers published between 2012 and the present were reviewed and classified as retrospective and prospective studies (Table 1 and Table 2). We reviewed 47 studies, 27 retrospective (Table 1) and 17 prospective (Table 2). We collected the number of male and female participants to quantify and analyze gender distribution in these studies. With this information, we calculated the number of male and female patients studied per year (Figure 1) in both the retrospective and prospective settings. We observed female underrepresentation in glioblastoma studies, both at the retrospective and prospective level, with an average of 41% and 37% female inclusion, respectively (Table 1 and Table 2), although it should be noted that several studies report distributions that more closely emulate male vs. female tumor incidence in glioma, whereas others suffer from more flagrant imbalances. There was little increase in female representation from 2012 to the present (Figure 1). Women are not sufficiently included in mixed-sex GBM trials to reflect the disease prevalence among the general population, given the observed range of 20–75% in retrospective trials and 0–67% in prospective trials (Table 1 and Table 2). This underrepresentation is reflected in prominent glioma landmark trials, as evidenced in a recent study that independently validates sex-specific prognostic nomograms [3] based on the original NRG/RTOG 0525 and 0825 clinical trials. Even when male/female inclusion is more balanced (57.7% vs. 60.3% males (NRG/RTOG 0525) and 42.3% vs. 39.7% females (NRG/RTOG 0825), clinical features between genders may remain unbalanced. The authors found that the age at diagnosis, performance status, MGMT methylation status, the extent of resection, use of corticosteroids, and location of the tumor in the brain were significant predictors of OS for males. However, in contrast, the extent of resection was not a significant predictor of OS for females. The authors attributed this to a proportion of female patients where resection status was captured as “other” [3]. The authors also noted that the relative importance of clinical covariates in the nomogram was different between sexes with age at diagnosis, MGMT methylation status, and performance status, which was higher for males compared to females, indicating worse survival for males compared to females. Such conclusions are hypothesis-generating but remain challenging to replicate given data limitations. The question of clinical trial representation was discussed in the context of the inclusion of women in clinical trials used for drug registration [19], with the conclusion that there was no underrepresentation of women. However, it has been pointed out that underrepresentation may exist in phase 1 and 2 trials, while being addressed in phase 3 [20]. This is a concern in glioma since potential sex differences associated with clinical benefits or increased toxicity may be missed in small, unbalanced cohorts. Retrospective studies (Table 1) may provide an avenue for more balanced data sets, given that they can include larger numbers of patients than most prospective studies (Table 1 and Table 2). However, many retrospective studies also report on small numbers of patients [21,22,23,24,25,26,27,28,29,30], wherein women make up roughly a third of the cohort, and larger studies [31,32,33,34,35,36,37,38,39,40] do not report on separate analyses for men and women to identify potential sex differences. Prospective studies (Table 2) often involve novel interventions and smaller patient numbers. Examples include Sanai et al. studying AZD1775 (20 patients) [41], Geltneky et al. studying oncolytic H-1 parvovirus (18 patients) [42], Wick et al. studying BAY1436032 in IDH-mutant solid tumors (4 patients) [43], Chinnaiyan et al. studying vorinostat plus bevacizumab (19 patients) [44]. However, similar to retrospective studies in larger trials, there are limited reports on gender differences [45,46,47,48]. Nonetheless, some large retrospective cohorts did identify sex-specific differences of note (Table 3) [6,7,10,49]. This leads to further examination of data embedded in large-scale data sets, as discussed in the following section.

## 3. Large-Scale Data Sets and Male/Female Representation

**Figure 2:**
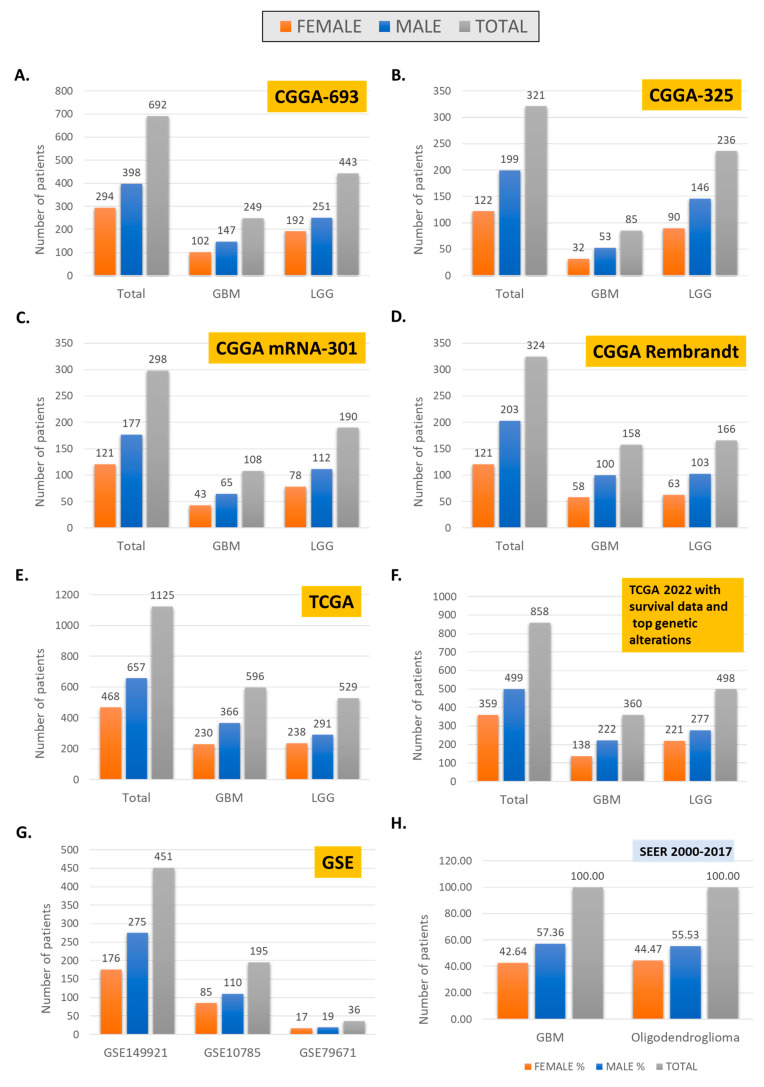
Gender distribution in large-scale omic data sets, CGGA (**A**–**D**), TCGA (**E**,**F**), GSE (**G**), and SEER (**H**).

Large-scale data sets may originate in registries or trials investigating specific therapeutic interventions. Exploration of sex differences in large-scale data is increasingly highly relevant, particularly with the improved capability of obtaining vast data sets from a relatively small number of samples, as with genomic, transcriptomic, and proteomic panels. Notable large databases include SEER [66], TCGA [67], CGGA [68], and several other evolving repositories (Table 4). These repositories are instrumental in advancing the field. However, they require orientation to provide detail for features selected for inclusion in analyses, as they exhibit significant heterogeneity in the proportion of histologies, number and type of features captured, and gender makeup (Table 4 and Figure 2). These aspects can result in nontransferable results when not corrected for and examined through a clinical lens. In a recent systematic review investigating validation of preclinical models employing 14 studies published between 2017 and 2020 using TCGA RNA microarray data, at least five biomarkers with discrepancies were identified, with only 29.4% of studies employing sex as a covariate, identical to MGMT methylation status [18]. Data sharing and collaborative data curation of multi-institutional data sets remain challenging. As a result, it is expected that data originating in large-scale registries compared to databases originating from clinical trial data may produce different conclusions, partly due to varying gender distribution and the impact of sex differences in molecular and management features. However, it should also be noted that other variables are significant in addition to gender, including comorbidities and molecular features. In a recent study aimed at generating sex-specific nomograms based on two significant large glioma trials, most patients included in the analysis had no comorbidities (45.9%), and patients with unknown methylation status were excluded from the analysis [3]. These aspects have intersectionality with gender and impact outcomes with significant confounders, many of which may be impossible to define given available data. Large-scale omics registries may also have missing values for molecular features, which diminishes the numbers available for analysis (Figure 2 panel E and F as of TCGA), and lack capture of comorbidities or performance status, which impact survival.

For example, data on all patients with GBM reported to the Swedish National Quality Registry for Primary Brain Tumors revealed that women had worse preoperative performance than males. For women with radical surgery, overall survival was improved. However, a survival advantage for women was no longer statistically significant in multivariate analysis, including of sex, age, surgery, and performance status [10]. Intersectionality with age is revealed in several studies, including in a recent analysis of brainstem tumors from the Surveillance, Epidemiology, and End Results (SEER) database between 2004 and 2018 [6], which revealed that, in younger patients, females had a higher age-adjusted mortality rate compared to males, with the reverse trend noted in older patients. A similar trend was identified for gliomas in CBTRUS (data from the NPCR and SEER) from 2000 to 2017 [7]. The significant intersectionality observed in large-scale data sets may be unavoidable. Hence, computational analyses must involve mitigation strategies to develop transferrable conclusions [69]. The question is perhaps not whether the numbers of women and men are comparable to each other, but instead (1) whether they reflect real-world data as exemplified by SEER (Figure 2H), and (2) have parallel analyses been carried out to identify sex differences if present.

## 4. Omics and Biospecimen Analysis

Biospecimen analysis is key to defining sex differences in malignancy. In neuro-oncology, this is evident given the growth in publications wherein sex differences are explored using omic approaches. Retrospective and prospective studies have previously identified potential prognostic and predictive factors grounded in sex differences. Sex differences have been noted in anti-epileptic management [70], chemotherapy [12,71], and immunotherapy [11,17] (Table 3). Looking ahead at the potential future results of omic analyses will therefore involve looking back at current and previous trials wherein a biospecimen was/is being collected to examine where future conclusions may originate from (Table 5) and how this bedside data connects to bench data, including tissue culture and animal studies. However, data from ongoing studies (Table 5) is years away, and it is unclear how existing data sets originating from completed studies may be combined to elicit robust conclusions. Data from tissue culture and animal studies may help fill this void as clinical and omic data sets grow. However, bench data underreporting biological sex in cell lines and biospecimens remains a significant barrier [72], and the functional characterization of brain tumor cells concerning sex differences is evolving [14]. In preclinical data studies published in AJP-Cell Physiology in 2013 [72], 75% of articles did not specify the sex of the cells employed, with the remaining 20% male and 5% female; in addition, biological sex remains underreported in biospecimen studies [18,73]. When screening for novel anti-cancer drugs in male- and female-derived human cell lines, higher toxicity levels were identified in male-derived cells, emphasizing the importance of annotating cell origin [72]. In a recent analysis aimed at the characterization of brain tumor-initiating cells for glioblastoma (GBM) preclinical models, two male-derived GBM cell lines (QNS108 and QNS120) and one female-derived GBM cell line (QNS315) were found to grow more rapidly in female mice brains with one male-derived GBM cell line (QNS108) exhibiting decreased survival in female mice in comparison with male mice [14]. Of note, commonly employed glioblastoma cell lines U87 (used in over 2000 studies) and U251 (used in over 2000 studies) are male-derived cell lines [74,75]. Because of ongoing concerns with genetic drift and long-term culture, patient-derived cultures are being proposed [76]. The GBM Patient Derived Xenograft (PDX) database represents a tumor patient-derived xenograft model repository with multi-omic characterizations [77]. In parallel, evidence is mounting about biological differences based on sex in genomic data. A recent systematic review and meta-analysis lent further evidence about existing sexual dimorphism of the immune system as related to clinical outcomes in glioblastoma immunotherapy. Upon analyzing genomic data and clinical trials looking at the effect of sex on the immune system and GBM outcome following immunotherapy, the authors identified that females exhibited enriched immunological signatures on gene set enrichment analysis, which correlated with survival advantage as compared to males, particularly as related to vaccine-based immunotherapy [17]. An increasing quantity of data is emerging on the relationship between tumor mutational burden (TMB) and its role as a prognostic marker, and gender, with male and female patients exhibiting differential TMB in some cancers concerning prognosis [78]. Currently, however, this relationship in glioma is not well established.

Nonetheless, as the depth and richness of data increases, the number of cases with available information decreases, and sex differences may become more challenging to tease out, as evidenced by data sets (Figure 2B–D, and Table 4). Molecular classification is also lacking or uneven in many data sets. This likely reflects more profound disparities, as recently discussed by Wang et al., in bioethical implications of current practices of molecular diagnostics in neuropathology [79].

## 5. Conclusions

In this review, we aimed to explore the origins of data in this area to understand the representation of women in study samples, and to identify if uneven sex distribution continues to exist in present-day glioblastoma clinical trials and large-scale data, given current guidelines for study design and analysis aiming for diversity and inclusion. Further, the goal was to determine if existing data sets allow for meaningful biospecimen collection and omics analyses to identify sex differences. Having explored the literary landscape in glioma from 2012 to the present, we identified that data sets do suffer from uneven male/female distribution, lagging in representation and data analysis, which impacts conclusions being generated. The existing limitations of data sets, both preclinical and clinical, currently used to generate biospecimens for omic analysis, and the lack of transparency in cohort selection, results in many analyses being inconsistent and challenging to validate. The difficulties in data acquisition and analysis are twofold: (1) health disparities and underrepresentation of women in existing and emerging data sets; and (2) potentially smaller and difficult to characterize connections at the genomic and proteomic level, particularly when analyzed in combined data sets lacking statistical power. This is further complicated by the intersectionality of social and economic differences between sexes, combined with distinct side effects that women may experience due to treatments (e.g., thrombocytopenia secondary to temozolomide), which are more nuanced and challenging to robustly capture. With unequal distributions in data sets, both prospective and retrospective, there are concerns that understanding treatment and outcome-altering differences may continue to be delayed or missed. This can lead to novel treatments generating conclusions based on data from a more significant proportion of male participants and healthier participants able to obtain treatment in clinical trials. In the long term, this can undermine optimization (dosage-dependent or biological effect-dependent factors) of management for both sexes. Seeds being sown for future analyses must ensure adequate inclusive specimens in glioma capable of detecting and generating testable hypotheses to capitalize on sex differences that carry clinically and therapeutically meaningful conclusions for improved therapies. The ability to achieve inclusiveness has the potential to drive the understanding of cancer biology beyond glioma and improve the overall outcome for patients. Strategic study design and analysis must reach beyond gender inclusion in trials and biospecimen-driven analysis to parallel separate omic characterization of male and female cells, animals, and patients to harness potentially meaningful sex differences. Sex and gender aspects must be considered when investigating novel agents, especially pharmacokinetic and pharmacodynamic aspects, in addition to health care delivery and public health initiatives. Enumerating the molecular bases for sex differences in GBM is likely to reveal fundamental modulators of cancer risk and outcomes and guide specific components of precision medicine approaches to cancer treatments. At this time, data on sex differences in glioma are just emerging and, given that significant mechanistic and physiologic questions remain unanswered, there is a lack of in-depth understanding regarding the underlying biologic role for sex differences being observed with data undergoing evolution in this area. Guidelines for data capture and analysis must ensure that studies are appropriately designed to detect sex differences to conduct a parallel but separate analysis of male and female cells, animals, and patients to allow for the possibility of building representative data sets of all subtypes. Participants in clinical trials must accurately reflect the demographic profile of those who will likely receive treatment in the future. Sex bias in clinical trials and retrospective data sets lead to treatments with understudied efficacy in the neglected sex. Citing similarities in disease characteristics and incidence as reasoning for observed uneven gender distribution is no longer sufficient to overcome barriers to in-depth analyses, from bench to bedside and back.

## Figures and Tables

**Table 1:** Retrospective studies in glioma illustrating the number of patients included in the study with % female inclusion and generated conclusions.

Publication	Title	Number of Patients	Conclusions
Schaff et al., 2020 [30]	Characterization of MGMT and EGFR protein expression in glioblastoma and association with survival	51 patients 17 females (33%)	A weak association was seen between MGMT protein expression and promoter methylation. Quantification of MGMT protein expression was inferior to MGMT methylation for prognostication in GBM.
Tanguturi et al., 2017 [31]	Leveraging molecular data sets for biomarker-based clinical trial design in glioblastoma	233 patients 107 females (46%)	There were associations between GBM genomic subgroups and clinical or molecular prognostic covariates, demonstrating potential impacts on clinical trial design and interpretation.
Mata et al., 2020 [21]	Genetic and epigenetic landscape of IDH-wildtype glioblastomas with *FGFR3*-*TACC3* fusions	37 patients 15 females (40.50%)	Patients with FGFR3-TACC3 fusions demonstrated characteristic associated mutational, copy-number, and methylation profiles, and patients with F3T3-positive tumors had clinical outcomes slightly better than patients with F3T3-wildtype tumors.
Montemurro et al., 2021 [50]	Surgical outcome and molecular pattern characterization of recurrent glioblastoma multiforme: A single-center retrospective series	63 patients 24 females (38.10%)	This study confirmed the extent of resection (EOR) at first and at recurrence as a significant predictor of outcome in patients with recurrent GBM.
Se- Hyuk et al. 2018 [22]	Procarbazine and CCNU Chemotherapy for Recurrent Glioblastoma with MGMT Promoter Methylation	8 patients 4 females (50%)	The efficacy of procarbazine and CCNU chemotherapy is not satisfactory.
Bum-Sup et al., 2020 [32]	A Radiosensitivity Gene Signature and PD-L1 Status Predict Clinical Outcome of Patients with Glioblastoma Multiforme in The Cancer Genome Atlas Dataset	277 patients 112 females (40%)	Taken together, PD-L1-high-RR group could potentially benefit from radiotherapy combined with PD-1/PD-L1 blockade and angiogenesis inhibition.
Burgenske et al., 2019 [23]	Molecular profiling of long-term *IDH*-wildtype glioblastoma survivors	49 patients 17 females (35%)	Unique attributes were observed regarding altered gene expression and pathway enrichment. These attributes may be valuable prognostic markers and are worth further examination.
Nowosielski et al., 2018 [33]	Radiologic progression of glioblastoma under therapy—an exploratory analysis of AVAglio	299 patients 114 females (38%)	Progression of glioblastoma under therapy can be characterized radiologically. These radiologic phenotypes are influenced by treatment and develop differently over time with differential outcomes. Complete resolution of contrast enhancement during treatment is a favorable factor for outcome.
Patrizz et al., 2021 [34]	Tumor recurrence or treatment-related changes following chemoradiation in patients with glioblastoma: does pathology predict outcomes?	137 patients 48 females (35%)	Histopathologic findings following chemoradiation do not correlate with clinical outcomes. Such findings should be considered during patient management and clinical trial enrollment.
Fontanilles et al., 2020 [51]	Simultaneous detection of *EGFR* amplification and *EGFRvIII* variant using digital PCR-based method in glioblastoma	62 patients 28 females (45%)	The results highlight that the dPCR assay using LNA-hydrolysis probes allowed the simultaneous detection of the *EGFR* amplification and *EGFRvIII* variant and may be used routinely in patients treated for glioblastoma.
Massey et al., 2020 [52]	Image-based metric of invasiveness predicts response to adjuvant temozolomide for primary glioblastoma	90 patients 30 females (33%)	Factors like patient age, cycles of TMZ received, time to nadir volume, and tumor nodularity are associated with volumetric response during adjuvant TMZ in GBM patients receiving standard of care treatment. Most notably, nodular tumors have a cycle-dependent and more favorable image-based response to TMZ than diffuse tumors.
Galia et al., 2012 [26]	PARP-1 protein expression in glioblastoma multiforme	27 patients 13 females (48%)	*PARP-1* gene is expressed in GBM. This finding may be envisioned as an attempt to trigger apoptosis in this tumor and in many other malignancies. The presence of the protein exclusively at the nucleus further supports the function played by this gene in genome integrity maintenance and apoptosis. Finally, PARP-1 staining may be used as GBM cell marker.
Faria et al., 2020 [39]	Intranasal perillyl alcohol therapy improves the survival of patients with recurrent glioblastoma harboring mutant variant for *MTHFR rs1801133* polymorphism	100 patients 38 females (38%)	rGBM patients under POH-based therapy harboring hypermethylated phenotype and TT variant for *rs1801133* had longer survival.
Egaña et al., 2020 [40]	Methylation of MGMT promoter does not predict response to temozolomide in patients with glioblastoma in Donostia Hospital	334 patients 139 females (42%)	No association was detected between methylation of MGMT promoter and molecular markers such as ATRX, IDH, p53, and Ki67
Beije et al., 2015 [53]	Prognostic value and kinetics of circulating endothelial cells in patients with recurrent glioblastoma randomized to bevacizumab plus lomustine, bevacizumab single agent, or lomustine single agent. A report from the Dutch Neuro-Oncology Group BELOB trial	141 patients 55 females (39%)	CEC numbers increased during treatment with bevacizumab plus lomustine but not during treatment with either agent alone, suggesting that this combination induced the most significant vascular damage
Malmström et al., 2017 [35]	Postoperative neoadjuvant temozolomide before radiotherapy versus standard radiotherapy in patients 60 years or younger with anaplastic astrocytoma or glioblastoma: a randomized trial	144 patients 55 females (38%)	No advantage of NeoTMZ was noted for the overall study population or subgroup of GBM, while NeoTMZ resulted in 5 years longer median survival for patients diagnosed as AA.
Piccioni et al., 2019 [36]	Analysis of cell-free circulating tumor DNA in 419 patients with glioblastoma and other primary brain tumors	419 patients 160 females (38%)	Contrary to previous studies with very low yields, we found that half of PBT patients had detectable ctDNA with genomically targetable off-label or clinical trial options for almost 50%.
Håvik et al., 2012 [24]	*MGMT* promoter methylation in gliomas assessment by pyrosequencing and quantitative methylation-specific PCR	58 patients 27 females (47%)	*MGMT* promoter methylation analysis gives sufficient prognostic information to merit its inclusion in the standard management of patients with high-grade gliomas, and in this study, pyrosequencing came across as the better analytical method.
Wick et al., 2013 [25]	Enzastaurin before and concomitant with radiation therapy, followed by enzastaurin maintenance therapy, in patients with newly diagnosed glioblastoma without *MGMT* promoter hypermethylation	57 patients 21 females (37%)	PFS-6 missed the primary planned outcome of 55%. The secondary exploratory analysis of resection status of the different subgroups of patients with biopsies, partial resection, and complete resection demonstrates the strong prognostic influence of resection on overall survival.
Etcheverry et al., 2014 [37]	*DGKI* Methylation Status Modulates the Prognostic Value of *MGMT* in Glioblastoma Patients Treated with Combined Radio-Chemotherapy with Temozolomide	399 patients 161 females (40%)	The study results improve the conventional *MGMT* stratification of GBM patients receiving standard treatment. These results could help interpret published or ongoing clinical trial outcomes and refine patient recruitment in the future.
Weller et al., 2015 [38]	MGMT Promoter Methylation Is a Strong Prognostic Biomarker for Benefit from Dose-Intensified Temozolomide Rechallenge in Progressive Glioblastoma: The DIRECTOR Trial	105 patients 36 females (34%)	Temozolomide rechallenge is a treatment option for MGMT promoter-methylated recurrent glioblastoma. Alternative strategies need to be considered for patients with progressive glioblastoma without MGMT promoter methylation.
Mohan et al., 2021 [54]	Proton therapy reduces the likelihood of high-grade radiation-induced lymphopenia in glioblastoma patients: phase II randomized study of protons vs photons	84 patients 37 females (44%)	Sex, baseline ALC, and whole-brain V20 were the strongest predictors of G3+L for patients with GBM treated with radiation and temozolomide. PT reduced brain volumes receiving low and intermediate doses and, consequently, reduced G3+L.
Guan et al., 2021 [55]	Safety and efficacy of Hypofractionated stereotactic radiosurgery for high-grade Gliomas at first recurrence: a single-center experience	70 patients 30 females (43%)	Salvage HSRS showed a favorable outcome and acceptable toxicity for rHGG
Song et al., 2020 [27]	Initial experience with scalp sparing radiation with concurrent temozolomide and tumor treatment fields (SPARE) for patients with newly diagnosed glioblastoma	10 patients 2 females (20%)	Concurrent TTFields with scalp-sparing chemoradiation is a safe and feasible treatment option with limited toxicity.
Kaley et al., 2018 [28]	BRAF Inhibition in *BRAF*^V600^-Mutant Gliomas: Results From the VE-BASKET Study	24 patients 18 females (75%)	Vemurafenib demonstrated evidence of durable antitumor activity in some patients with *BRAF*^V600^-mutant gliomas, although efficacy seemed to vary qualitatively by histologic subtype.
Nishii et al., 2018 [29]	Differential Diagnosis between Low-Grade and High-Grade Astrocytoma Using System A Amino Acid Transport PET Imaging with C-11-MeAIB: A Comparison Study with C-11-Methionine PET Imaging	31 patients 15 females (48%)	MeAIB, a system A amino acid transport-specific radiolabeled agents, could provide better assessments for detecting malignant type brain tumors.
Biau et al., 2017 [56]	Radiotherapy plus temozolomide in elderly patients with glioblastoma: a “real-life” report	104 patients 51 females (49%)	These outcomes agree with the literature regarding optimal surgery and HFRT as a standard treatment for elderly GBM patients.

**Table 2:** Prospective studies in glioma illustrating the number of patients included in the study with % female inclusion and generated conclusions.

Publication	Title	Number of Patients	Conclusions
Sanai et al., 2018 [41]	Phase 0 Trial of AZD1775 in First-Recurrence Glioblastoma Patients	20 patients 8 females (40%)	In contrast to recent preclinical data, this phase 0 study of AZD 1775 in recurrent glioblastoma indicates good human brain tumor penetration, provides the first evidence of clinical, biological activity in human glioblastoma and confirms the utility of phase 0 trials as part of an accelerated paradigm for drug development in patients with glioma.
Geletneky et al., 2017 [42]	Oncolytic H-1 Parvovirus Shows Safety and Signs of Immunogenic Activity in a First Phase I/IIa Glioblastoma Trial	18 patients 4 females (22%)	The ParvOryx01 trial data confirm H-1PV safety and tolerability. This trial points to H-1PV capacity for establishing an immunogenic tumor microenvironment, making H-1PV an interesting candidate for further clinical development.
Wick et al., 2021 [43]	Phase I Assessment of Safety and Therapeutic Activity of BAY1436032 in Patients with IDH1-Mutant Solid Tumors	4 patients 0 females (0%)	BAY1436032 was well tolerated and showed evidence of target inhibition and durable objective responses in a small subset of subjects with LGG.
Chinnaiyan et al., 2012 [44]	Phase I trial of vorinostat combined with bevacizumab and CPT-11 in recurrent glioblastoma.	19 patients 7 females (37%)	Based on the intimate cross-talk and coregulation between VEGF and PDGF signaling, it can be hypothesized that continued VEGF inhibition may modulate PDGF-AA expression through regulatory feedback inhibition, thereby attenuating inhibitory signaling contributing toward VEGF-independent progression.
Cloughesy et al., 2017 [45]	Randomized, Double-Blind, Placebo-Controlled, Multicenter Phase II Study of Onartuzumab Plus Bevacizumab Versus Placebo Plus Bevacizumab in Patients with Recurrent Glioblastoma: Efficacy, Safety, and Hepatocyte Growth Factor and O^6^-Methylguanine-DNA Methyltransferase Biomarker Analyses	129 patients 46 females (36%)	There was no evidence of further clinical benefit with the addition of onartuzumab to bevacizumab compared with bevacizumab plus placebo in unselected patients with recurrent glioblastoma in this phase II study.
Maraka et al., 2020 [46]	Phase 1 Lead-in to a Phase 2 Factorial Study of Temozolomide Plus Memantine, Mefloquine, and Metformin as Postradiation Adjuvant Therapy for Newly Diagnosed Glioblastoma	107 patients 42 females (40%)	Memantine, mefloquine, and metformin can be combined safely with TMZ in patients with newly diagnosed glioblastoma.
Nabors et al., 2015 [47]	Two cilengitide regimens in combination with standard treatment for patients with newly diagnosed glioblastoma and unmethylated *MGMT* gene promoter: results of the open-label, controlled, randomized phase II CORE study	265 patients 110 females (42%)	Standard and intensive cilengitide dose regimens were well tolerated in combination with TMZ/RT→TMZ. Inconsistent overall survival and progression-free survival outcomes and limited sample size did not allow firm conclusions regarding clinical efficacy.
Omuro et al., 2014 [57]	Phase II Study of Bevacizumab, Temozolomide, and Hypofractionated Stereotactic Radiotherapy for Newly Diagnosed Glioblastoma	40 patients 14 females (35%)	This aggressive radiotherapy schedule was safe and more convenient for patients, achieving an OS comparable to historical controls. Analysis of advanced neuro-imaging parameters suggests ADC and FDG-PET as potentially valuable biomarkers, whereas tissue correlatives uncovered the poor prognosis associated with the proneural signature in non-IDH-1 mutated glioblastoma.
Miller et al., 2022 [58]	Immune activity and response differences of oncolytic viral therapy in recurrent glioblastoma: Gene expression analyses of a Phase IB study	6 patients 4 females (67%)	The data supports that the oHSV-induced type I IFN production and the subsequent recruitment of an adaptive immune response differed between enrolled patients and showed an association with survival duration in patients with recurrent malignant glioma after treatment with an early generation oHSV.
Thomas et al., 2017 [59]	Multicenter phase II study of temozolomide and myeloablative chemotherapy with autologous stem cell transplant for newly diagnosed anaplastic oligodendroglioma	41 patients 14 females (34%)	TMZ followed by HDC-ASCT can be safely administered to patients with newly diagnosed 1p/19q co deleted AO.
Norden et al., 2013 [60]	Phase 2 study of dose-intense temozolomide in recurrent glioblastoma	55 patients 22 females (40%)	Dose-intense temozolomide on this schedule is safe in recurrent GBM. However, efficacy is marginal and predictive biomarkers are needed.
Han et al., 2014 [61]	Phase II trial of 7 days on/7 days off temozolomide for recurrent high-grade glioma	60 patients 22 females (37%)	The dose-dense temozolomide regimen was well tolerated, although it has no significant activity in this population.
Herrlinger et al., 2016 [48]	Bevacizumab Plus Irinotecan Versus Temozolomide in Newly Diagnosed O6-Methylguanine-DNA Methyltransferase Non methylated Glioblastoma: The Randomized GLARIUS Trial	170 patients 56 females (33%)	BEV+IRI resulted in a superior PFS-6 rate and median PFS compared with TMZ. However, BEV+IRI did not improve OS, potentially because of the high crossover rate. BEV+IRI did not alter QOL compared with TMZ.
Wick et al., 2016 [62]	Phase II Study of Radiotherapy and Temsirolimus versus Radiochemotherapy with Temozolomide in Patients with Newly Diagnosed Glioblastoma without MGMT Promoter Hypermethylation (EORTC 26082)	111 patients 40 females (36%)	Temsirolimus was not superior to temozolomide in patients with an unmethylated MGMT promoter. Phosphorylation of mTORSer2448 in the pretreatment tumor tissue may define a subgroup benefitting from mTOR inhibition.
Lombardi et al., 2015 [63]	Clinical and Genetic Factors Associated with Severe Hematological Toxicity in Glioblastoma Patients During Radiation Plus Temozolomide Treatment: A Prospective Study	87 patients 32 females (37%)	Although we studied a small population, we suggest clinical and genetic factors might simultaneously be associated with severe myelosuppression developed during TMZ plus RT.
Pitz et al., 2015 [64]	Phase II study of PX-866 in recurrent glioblastoma	33 patients 12 females (36%)	PX-866 was relatively well tolerated. The overall response rate was low, and the study did not meet its primary endpoint; however, 21% of participants obtained durable, stable disease.
Hu et al., 2013 [65]	A phase II trial of oral gimatecan for recurrent glioblastoma	29 patients 17 females (59%)	Treatment with gimatecan 1.0 mg/m2/day for 5 days, repeated every 28 days, showed minimal efficacy.

**Table 3:** Studies aimed at sex-specific outcome differences in glioma illustrating the number of patients included in the study with % female inclusion and generated conclusions.

Publication	Title	Cohort Origin	Number of Patients	Conclusion
Tewari et al., 2022 [49]	Sex-Specific Differences in Low-Grade Glioma Presentation and Outcome	Single institution	372/792 (47% female) 291 with molecularly avail status	Female sex independently associated with improved outcomes in pts with avail molecular status.
Tavelin et al., 2022 [10]	Sex Differences in Glioblastoma-Findings from the Swedish National Quality Registry for Primary Brain Tumors between 1999–2018	Swedish National Quality Registry for Primary Brain Tumors	2083/5243 (40% female)	Sex-related differences in clinical factors could be identified in a population-based cohort. In this data set, for survival, the only advantage noted was for women who had undergone radical surgery, although this was clinically almost negligible.
Tomita et al., 2021 [6]	Fifteen-year trends and differences in mortality rates across sex, age, and race/ethnicity in patients with brainstem tumors	SEER (2004–2018)	395/838 (47%) female (younger< 14 yro) 520/1201 (43%) female (older> 15 yro)	The age-adjusted mortality rate is higher for 5–9 years of age, with a reverse trend seen for 50–79 years of age.
Wang et al., 2022 [7]	Importance of the intersection of age and sex to understand variation in incidence and survival for primary malignant gliomas	CBTRUS (data NPCR and SEER)	130 051/294 886 (44.1%) female	Females had worse survival for ages 0–9, male survival worse for all other age groups, with the difference highest in 20–29 years.

**Table 4:** Large scale multi-channel data repositories with gender capture parameters [67,68].

Data Set	Molecular Data	Samples #	Gender Distribution	GBM/LGG Samples #
CGGA—693 mRNA—RNAseq	mRNA	693	Total—693 Male—398 Female—295	GBM—249 LGG—443
CGGA—mRNA 325 samples RNAseq	mRNA	325	Total—325: Male—203 Female—122	GBM—109 LGG—212
CGGA—mRNA microarray 301 samples	mRNA	301	Total—301 Male—180 Female—121	GBM—113 LGG—185
	mRNA			
CGGA—miRNA micro-array 198	miRNA	198	Total—198 Male—123 Female—75	GBM—85 LGG—113
CGGA—methylation micro-array	Methyl	159	Total—159 Male—89 Female—62 NA—8	GBM—43, Normal—8 LGG—108
CGGA—Mutation Data	mutation	286	Total—286 Male—168 Female—118	GBM—102 LGG—184
CGGA—Normal RNA-seq	mRNA	20	NA	Normal—20
CGGA—Rembrandt mRNA Array 475 samples	mRNA	475	Total—475: Male—203 Female—121 NA—151	GBM—183 LGG—221
TCGA—LGG	mRNA	530	Total—530 Male—291 Female—238	LGG—530
	meth	530		LGG—530
	miRNA	526		LGG—526
	CNV	514		LGG—514
TCGA—GBM	mRNA	166	Total—595 Male—365 Female—230	GBM—166
	meth	285		GBM—285
	miRNA	5		GBM—5
	CNV	595		GBM—595

**Table 5:** Glioma trials aimed at biomarker identification with estimated enrollment exceeding 100 participants [80].

Study	Number of Patients	Anticipated Completion
ALBATROSS Study: International Multicenter Study for Prospective Validation of Imaging Biomarkers Calculated at Vascular Habitats of High-grade Gliomas (ALBATROSS)	300	1 June 2022
The circTeloDIAG: Liquid Biopsy for Glioma Tumor (circTeloDIAG)	150	August 2023
Visual Study of Molecular Genotype in Glioma Evolution	1000	31 December 2021
Glioma Brain Tumours-E12513-SensiScreen Glioma	220	31 December 2022
Studying the Biology of IDH-mutant Gliomas Via Longitudinal Observation of 2-hydroxyglutarate (2-HG) Using MR Spectroscopy	270	31 December 2025
Survival Significance of Molecular Pathology and Genetic Variation in Brain Gliomas	3000	1 January 2025
Evaluating the Expression Levels of MicroRNA-10b in Patients With Gliomas	200	May 2022

## Data Availability

Data supporting reported results is public and can be found in alphabetical order at: CGGA (Chinese Glioma Genome Atlas) (http://www.cgga.org.cn/, accessed on 25 July 2022); ClinicalTrials.gov. (https://clinicaltrials.gov/ct2/results/, accessed on 25 July 2022); PubMed (https://pubmed.ncbi.nlm.nih.gov//, accessed on 25 July 2022); SEER (The Surveillance, Epidemiology, and End Results)(SEER) Program. (https://seer.cancer.gov//, accessed on 25 July 2022); TCGA (The Cancer Genome Atlas Program) (https://portal.gdc.cancer.gov//, accessed on 25 July 2022).

## Abbreviations

CBTRUS	Central Brain Tumor Registry of the United States
CGGA	Chinese Glioma Genome Atlas +CNS Central Nervous System
GBM	Glioblastoma Multiforme
MGMT	Management Science Replication Project.
NPCR	National Program of Cancer Registries
PDX	Patient-Derived Xenograft
SEER	Surveillance, Epidemiology, and End Results
TCGA	The Cancer Genome Atlas
TCIA	The Cancer Imaging Archive
TMB	Tumor Mutational burden
